# Supplementary material for: How Easy Is It to Learn Motion Models from Widefield Fluorescence Single Particle Tracks?
Source: ArXiv. 2025 Jul 25:arXiv:2507.05599v3. Preprint. [Version 3] (PMC12265587)
Supplement: Supplement 1 [file NIHPP2507.05599v3-supplement-1.pdf]

Supplementary Information

Contents

References (Main) **15**

Part I: Burn In Removal **20**

Part II: Alternate Motion Models for Anomalous Diffusion **21**
Directed Brownian Motion 21
Fractional Brownian Motion 21
Lévy Walks 22
Scaled Brownian Motion 23

Part III: Alternate Emission Models **23**
Emission Model for Poisson-Gamma-Normal (PGN) Noise Model of EMCCD Detectors 23
Emission Model for sCMOS Detectors 24

Part IV: Supplementary Figures **25**
Motion Models and Anomalousness 25
Tracking Non-Brownian Motion 26

Part V: Supplementary Tables **28**

References (Supplementary) **28**

Part I: Burn In Removal

Markov Chain Monte Carlo (MCMC) samplers are often started from an intentionally over-dispersed initial guess — a point in the parameter space that is unlikely to coincide with the data-driven mode. Accordingly, the early search resembles a biased exploration, and this burn in (*i.e.*, initialization bias) should be removed to ensure that posterior summaries reflect equilibrium sampling. In SI Figure 4 below, we show inferred values of diffusivity $\mathfrak{D}$ from $I = 2000$ MCMC samples. Since the first $\approx 50\%$ of samples are non-stationary, we keep only the second half of samples $i \in [1, 2] \cdot 10^3$; consequently, the mean over kept iterations adequately represents the high posterior density and approximates ground truth.

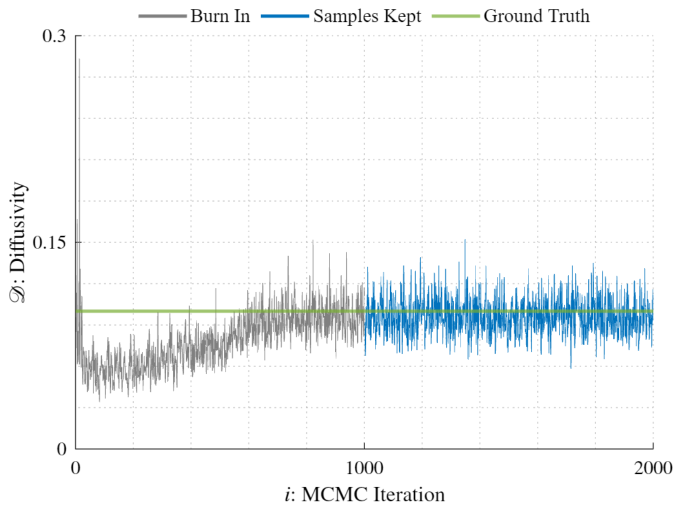

SI Figure 4: Inferred diffusivity $\mathcal{D}$ ($\mu m^2/s$) for a single Brownian walker from an MCMC chain of $I = 2000$ iterations. Samples from the initial 999 iterations (gray) constitute the burn-in phase; they display a clear upward drift as the chain moves from its over-dispersed starting point toward the high-posterior-density region. The remaining 1000 iterations (blue) fluctuate symmetrically around the ground truth $\mathcal{D}$ (green), indicating that the chain has reached stationarity. Discarding the burn-in therefore removes initialization bias while preserving samples that faithfully represent the target posterior distribution.

Part II: Alternate Motion Models

Here, we present additional motion models that can be incorporated into our likelihood. In what follows, we describe the defining features and probabilistic formulations for each candidate model. Because the formulations for FBM, LW, and SBM are intended for equally spaced lag times, interpolation is not advised; therefore, L should be defined as the number of observed frames (N in the main text).

DBM Motion Model

Directed Brownian motion (DBM) follows the same stochastic framework as Brownian motion (BM) model (Equation 3 in the main text) — namely, a sequence of Brownian (*i.e.*, statistically independent, stationary, Gaussian) transitions. However, this motion model allows for a constant drift speed v that biases each displacement in a fixed direction $\hat{\boldsymbol{v}}$. Accordingly, the evolution of the particle's position is governed by both stochastic diffusion and deterministic drift, with the resulting motion model given by

$$\mathbb{P}(\Delta \boldsymbol{R}_{1:L}) = \exp\left[-\sum_{\ell=1}^{L} \frac{|\Delta \boldsymbol{R}_\ell - \boldsymbol{v}\Delta t_\ell|^2}{4\mathcal{D}\Delta t_n^k}\right] \prod_{\ell=2}^{L} (4\pi\mathcal{D}\Delta t_\ell)^{-3/2}, \tag{19}$$

where $\boldsymbol{v} \equiv v\hat{\boldsymbol{v}}$ is the particle's velocity vector, $\Delta t_\ell \equiv t_\ell - t_{\ell-1}$ is the lag-time between particle positions spanning a Euclidean distance of $|\Delta \boldsymbol{R}_\ell - \boldsymbol{v}\Delta t_\ell|$.

FBM Motion Model

We generalize the one-dimensional motion model[1,2] for FBM to three-dimensional space as

$$\mathbb{P}(\Delta \boldsymbol{R}_{1:L}) = \left[(2\pi)^L |\boldsymbol{\Sigma}_L|\right]^{-\frac{3}{2}} \exp\left[-\frac{1}{2}\sum_{\ell=1}^{L} \Delta \boldsymbol{R}_\ell^\top \left(\boldsymbol{\Sigma}_L^{-1} \otimes \mathbb{I}\right) \Delta \boldsymbol{R}_\ell\right], \tag{20}$$

where $\boldsymbol{\Sigma}_L$ is the covariance matrix with elements $(\Sigma_L)_\ell^{\ell'} = \gamma(\ell - \ell')$ defined through the autocovariance function

$$\gamma(\ell) = \mathcal{D}_{\mathcal{H}} t_E^{2\mathcal{H}} \left[|\ell+1|^{2\mathcal{H}} + |\ell-1|^{2\mathcal{H}} - 2|\ell|^{2\mathcal{H}}\right], \tag{21}$$

and $\otimes$ denotes the Kronecker product. Because inversion of $\mathbf{\Sigma}_L$ is computationally expensive, we explore a second means by which SI Equation 20 can be calculated: the likelihood can be rewritten using the chain rule of conditional probabilities:

$$\mathbb{P}(\varDelta\boldsymbol{R}_{1:L}) = (2\pi)^{-\frac{3L}{2}} \prod_{\ell=1}^{L} \frac{1}{\varsigma_\ell^3} \exp\left[-\frac{(\varDelta\boldsymbol{R}_\ell - \varDelta\boldsymbol{\mu}_\ell)^2}{2\varsigma_\ell^2}\right], \tag{22}$$

where the mean $\boldsymbol{\mu}$ and standard deviation $\boldsymbol{\varsigma}$ are iterated using the Durbin-Levinson algorithm [1, 3]. For iteration of the mean and standard deviation, we initialize $\varDelta\boldsymbol{\mu}_1 = \mathbf{0}$ and $\sigma_1^2 = \gamma(0)$ before recursively iterating successive values through

$$\begin{cases} \varDelta\boldsymbol{\mu}_{\ell+1} & = \sum\limits_{\ell'=1}^{\ell} \phi_\ell^{\ell'}\, \varDelta\boldsymbol{R}_{\ell+1-\ell'}, \\ \varsigma_{\ell+1}^2 & = \varsigma_\ell^2 \left[1 - (\phi_\ell^\ell)^2\right], \end{cases} \tag{23}$$

where the coefficients $\phi_\ell^{\ell'}$ are obtained using SI Equation 21 as

$$\begin{cases} \phi_1^1 = \gamma(1)/\gamma(0) \\ \phi_\ell^\ell = \sigma_\ell^{-2}\, \gamma(\ell) - \sum\limits_{\ell'=1}^{\ell-1} \gamma(\ell - \ell')\, \phi_{\ell-1}^{\ell'}, \\ \phi_\ell^{\ell'} = \phi_{\ell-1}^{\ell'} - \phi_{\ell-1}^{\ell-\ell'}\, \phi_\ell^\ell, \quad 1 \le \ell' < \ell. \end{cases} \tag{24}$$

Prior Distributions: Since the Hurst parameter exists along the interval $\mathscr{H} \in (0,1)$, modeling it as a uniformly distributed hyperparameter gives its distribution as $\mathbb{P}(\mathscr{H}) = 1$. To then assign prior knowledge to the anomalous diffusivity, we prescribe a Jeffreys' prior[4] on the standard deviation for a single step $\sigma_{\mathscr{H}}$:

$$\mathbb{P}(\sigma_{\mathscr{H}}) = \begin{cases} \left(\sigma_{\mathscr{H}} \ln \dfrac{\overline{\sigma}_{\mathscr{H}}}{\underline{\sigma}_{\mathscr{H}}}\right)^{-1} & , \quad \sigma_{\mathscr{H}} \in [\underline{\sigma}_{\mathscr{H}}, \overline{\sigma}_{\mathscr{H}}] \\ 0 & , \quad \sigma_{\mathscr{H}} \notin [\underline{\sigma}_{\mathscr{H}}, \overline{\sigma}_{\mathscr{H}}]. \end{cases} \tag{25}$$

Doing so allows us to define the generalized diffusivity[1] as

$$\mathscr{D}_{\mathscr{H}} = \frac{\sigma_{\mathscr{H}}^2}{2t_E^{2\mathscr{H}}}. \tag{26}$$

LW Motion Model

Although there exists a one-dimensional motion model for LW[1], it was parameterized upon an *ad hoc* global step deviation. Accordingly, we must first re-parameterize the model to account for realistic static and dynamic localization errors. From known localization formulae[5], we consider the static and dynamic localization error to manifest as

$$\varsigma_\ell^p = \sqrt{\frac{\sigma_{xy}^2 + \varDelta s^2/12}{\widetilde{C}_\ell^p} + \frac{8\pi\sigma_{xy}^4 (B_\ell^p)^2}{(\widetilde{C}_\ell^p)^2 \varDelta s^2} + \frac{\sigma_{\text{read}}^2}{(\widetilde{C}_\ell^p)^2} + \frac{v^2 \varDelta t_\ell^2}{12}}, \tag{27}$$

where $\widetilde{C}_\ell^p \equiv \beta(G/\varphi)u_\ell^p$ represents the photons counted at the pth pixel in the ℓth frame from photoelectron load u_ℓ^p apportioned by quantum efficiency β, dimensionless gain G, and the calibration parameter φ; σ_{xy} is the PSF's axial width, $\varDelta s$ is the pixel side length, $(B_\ell^p)^2 = \beta(G/\varphi)^2 F A^p \varDelta t_\ell + \sigma_{\text{read}}^2$ is the constant background noise, and $v^2\varDelta t_\ell^2/12$ is a Lévy walker's dynamic localization error for constant speed v over the exposure time $\varDelta t_\ell \equiv t_E$. If we constrain our investigation to EMCCDs operated at high gain G, then background noise is dominated by Poissonian photon statistics such that the readout noise can be neglected: $B_\ell^2 \approx G^2 F A t_E$. Having defined the standard deviation of localization in the ℓth frame as ς_ℓ through SI Equation 27, we now prescribe the LW motion model for experimental imaging data as

$$\mathbb{P}(\varDelta\boldsymbol{R}_{1:L}) = \prod_{\ell=1}^{L} \prod_{p=1}^{P} \left[4\pi(\varsigma_\ell^p)^2\right]^{-\frac{3}{2}} \exp\left(-\frac{|\varDelta\boldsymbol{R}_\ell - v t_E \hat{\boldsymbol{v}}_\ell|^2}{4(\varsigma_\ell^p)^2}\right) \tag{28}$$

for jump directions sampled on the the unit sphere $\hat{\boldsymbol{v}}_\ell \sim \mathbb{U}[\mathbb{S}]$.

Prior Distributions: Since LW only models superdiffusion and ballistic diffusion, the anomalous exponent is constrained to exist along the interval $\alpha \in (1, 2]$; modeling α as a uniformly distributed hyperparameter, then, gives

$\mathbb{P}(\alpha) = 1$. Given that random jumps of length vt_E with constant velocity $v \sim \mathbb{U}_{[0,10]}$ were sampled on the the unit sphere $\hat{\boldsymbol{v}} \sim \mathbb{U}[\$]$, we need only further address the radial direction associated with each jump. That is, we adopt the radial vector described by length $r = vt_E$, azimuth $\phi \sim \mathbb{U}_{[0,2\pi]}$, and zenith $\theta \sim \mathbb{U}_{[0,\pi]}$. Accordingly, our joint prior is written $\mathbb{P}(\alpha)\,\mathbb{P}(v)\,\mathbb{P}(\theta)\,\mathbb{P}(\phi) = \frac{1}{20\pi^2}$.

SBM Motion Model

We generalize SBM's motion model[2] to

$$\mathbb{P}(\varDelta\boldsymbol{R}_{1:L}) = (2\pi)^{-\frac{3L}{2}} \prod_{\ell=1}^{L} \frac{1}{\varsigma_\ell^3} \exp\left(-\frac{|\varDelta\boldsymbol{R}_\ell|^2}{2\varsigma_\ell^2}\right), \tag{29}$$

where τ is the system's aging prior to $t = 0$, and the time-dependent standard deviation for a single step is

$$\varsigma_\ell^2 = 2\mathscr{D}_\alpha t_E^\alpha \left[\left(\ell + \frac{\tau}{t_E}\right)^\alpha - \left(\ell - 1 + \frac{\tau}{t_E}\right)^\alpha\right]. \tag{30}$$

Prior Distributions: Since SBM describes subdiffusion, BM, superdiffusion, and ballistic diffusion, we model the anomalous exponent as a uniformly distributed hyperparameter along the interval $\alpha \in (0, 2]$, which returns its distribution as $\mathbb{P}(\alpha) = 1/2$. Following the one-dimensional formulation[2], we prescribe a standard normal distribution $\mathbb{N}(0, 1)$ on $\log_{10} \varsigma_1$ such that $\mathbb{P}(\varsigma_1) = (\varsigma_1 \ln 10\sqrt{2\pi})^{-1} \exp\left[-(\log_{10} \varsigma_1)^2/2\right]$.

Part III: Alternate Emission Models

EMCCD Poisson-Gamma-Normal (PGN) Noise Model

Step 1: Photon Arrival & Photoelectron Genesis

Light captured by a detector comes from both signal and noise. The incident photon flux per pixel per frame, given a photon emission rate H contributing to fluorophore signal in an environment of ambient photon flux F contributing to background illumination, is

$$\varPhi_n^p \equiv [F + H\,\mathrm{PSF}(x^p, y^p\,;\,\boldsymbol{R}_n)]\;\mathrm{d}A^p\,\mathrm{d}t_n, \tag{31}$$

where $\mathrm{d}t_\ell \equiv t_E$ is merely the exposure period, and $\mathrm{d}A^p$ is the area of the pth pixel. Upon capturing this light, $\varPhi_n^p$ gets apportioned by the detector's quantum efficiency β characterizing its ability to generate a photoelectron per incident photon. Additionally, thermal noise manifests as "dark" photoelectron counts $\not{C}_n^p \equiv \dot{\not{C}}_n^p\,\mathrm{d}A^p\mathrm{d}t_n$ arising from the dark current $\dot{\not{C}}^p$ over area $\mathrm{d}A^p$ in duration t_E. Furthermore, the clock-induced charge (CIC) c spuriously generates electrons, adding an exposure-independent Poisson offset to pixels. Since any source of light consists of discrete quanta, the detector's pre-amplification photoelectron load C_n^p in frame n at pixel p is Poisson distributed:

$$\mathbb{P}(C_n^p \mid \varPhi_n^p) = \frac{(\beta\,\varPhi_n^p + \dot{\not{C}}^p t_E + c)^{C_n^p}}{C_n^p!} \exp\Big[-(\beta\,\varPhi_n^p + \dot{\not{C}}^p t_E + c)\Big]. \tag{32}$$

Step 2: Stochastic Multiplication in EM Register

In an EMCCD camera, photoelectrons comprising the pre-load C_n^p traverse an electron-multiplying (EM) gain register at random, yielding a post-amplification photoelectron load C'^p_n that follows Tubb's distribution

$$\mathbb{P}\big(C'^p_n \,\big|\, C_n^p\big) = \frac{(C'^p_n - C_n^p + 1)^{C_n^p - 1}}{(C_n^p - 1)!(G + 1/C_n^p - 1)^{C_n^p}} \exp\left(-\frac{C'^p_n - C_n^p + 1}{G + 1/C_n^p - 1}\right), \tag{33}$$

where G is the gain and $0 < C_n^p \le C'^p_n$. This stage introduces the multiplicative noise with an exponential tail that is characteristic of EMCCDs[6].

Step 3: Analogue Read Out

During read-out, the amplified photoelectron load ${C'}_n^p$ gets converted to voltage before being digitized as an analog-to-digital unit (ADU) count w_n^p. This read-out process introduces a zero-mean Gaussian noise with standard deviation σ. Thus, the probability distribution of raw measurements w_n^p is read

$$\mathbb{P}\big(w_n^p \,\big|\, {C'}_n^p\big) = (2\pi)^{-\frac{1}{2}} \frac{1}{\sigma^p} \exp\left[-\frac{(w_n^p - G^p {C'}_n^p)^2}{2(\sigma^p)^2}\right]. \tag{34}$$

The PGN Emission Model

Propagating uncertainties from photon statistics, stochastic multiplication, and the final output entails convolving the Poisson, EM-gain, and read-out stages. This Poisson-Gamma-Normal (PGN) noise model reads

$$\mathbb{P}\Big(w_{1:N}^{1:P} \,\Big|\, C_{1:N}^p, {C'}_{1:N}^{1:P}\Big) = \prod_{n=1}^{N}\prod_{p=1}^{P}\left[\sum_{C_n^p=0}^{\infty}\sum_{{C'}_n^p=0}^{\infty} \mathbb{P}(C_n^p \mid \Phi_n^p)\, \mathbb{P}\big({C'}_n^p \,\big|\, C_n^p\big)\, \mathbb{P}\big(w_n^p \,\big|\, {C'}_n^p\big)\right]. \tag{35}$$

SI Equation 35 above replaces Equation 6 in the main text for any values of w_n^p, G, and σ^p; the sums can be truncated at sufficiently high counts for a satisfactory approximation.

sCMOS Detector Architecture

Step 1: Photon Arrival & Photoelectron Genesis

Once more, we define the incident photon flux per pixel per frame from SI Equation 31 as

$$\Phi_n^p \equiv [F + H\, \mathrm{PSF}(x^p, y^p\,;\, \boldsymbol{R}_n)]\; \mathrm{d}A^p\, \mathrm{d}t_n,$$

where H is the photon emission rate contributing to fluorophore signal in an environment of ambient photon flux F contributing to background illumination; $\mathrm{d}A^p$ is the area of the pth pixel and $\mathrm{d}t_\ell \equiv t_E$ is the detector's exposure period. Since detectors generate photoelectrons in proportion to the light quanta (*i.e.*, photons) arriving randomly at the pth pixel in the nth frame, the photoelectron pre-load C_n^p is Poisson distributed with its rate apportioned by the quantum efficiency β:

$$\mathbb{P}(C_n^p \mid \Phi_n^p) = \frac{(\beta\,\Phi_n^p)^{C_n^p}}{C_n^p!} \exp\left(-\beta\,\Phi_n^p\right). \tag{36}$$

Step 2: Analog-to-Digital Conversion

Detectors of sCMOS architecture vary from pixel-to-pixel; such detectors are thusly characterized by pixel-dependent gain G^p, offset (bias) O^p, and read-noise variance $(\sigma^p)^2$. For such a detector, photoelectrons C_n^p are converted into an analog voltage before being digitized to a raw measurement value w_n^p in ADU[7]:

$$\mathbb{P}(w_n^p \mid C_n^p) = (2\pi)^{-\frac{1}{2}} \frac{1}{\sigma^p} \exp\left[-\frac{(w_n^p - O^p - G^p C_n^p)^2}{2(\sigma^p)^2}\right]. \tag{37}$$

The sCMOS Emission Model

Marginalizing the Poisson distributed photoelectron count C_n^p out of the Poisson-Gaussian process yields an infinite Gaussian mixture — the exact likelihood of recording w_n^p given Φ_n^p:

$$\mathbb{P}\big(w_{1:N}^{1:P} \mid \Phi_{1:N}^{1:P}\big) = \prod_{n=1}^{N}\prod_{p=1}^{P}\sum_{j=0}^{\infty} \frac{(\Phi_n^p)^j}{j!\sqrt{2\pi(\sigma^p)^2}} \exp\left[-\Phi_n^p - \frac{(w_n^p - O^p - jG^p)^2}{2(\sigma^p)^2}\right]. \tag{38}$$

For detectors of sCMOS architecture, SI Equation 38 stands in place of Equation 6 in the main text for any values of Φ_n^p. With modest illumination (*i.e.*, $\Phi_{1:N}^{1:P} \geq 5$ photons), the Poisson term in SI Equation 38 can be approximated by a Gaussian of variance Φ_n^p without altering the emission model by more than 1%[8]; this approximation yields the closed-form

$$\mathbb{P}(w_{1:N}^{1:P} \mid \Phi_{1:N}^{1:P}) \approx (2\pi)^{-\frac{NP}{2}} \exp\left[-\sum_{n=1}^{N}\sum_{p=1}^{P} \frac{(w_n^p - O^p - G^p\,\Phi_n^p)^2}{2\left[(G^p)^2\,\Phi_n^p + (\sigma^p)^2\right]}\right] \prod_{n=1}^{N}\prod_{p=1}^{P} \left\{\left[(G^p)^2\,\Phi_n^p + (\sigma^p)^2\right]\right\}^{-\frac{1}{2}}. \tag{39}$$

Part IV: Supplementary Figures

Motion Models and Anomalousness

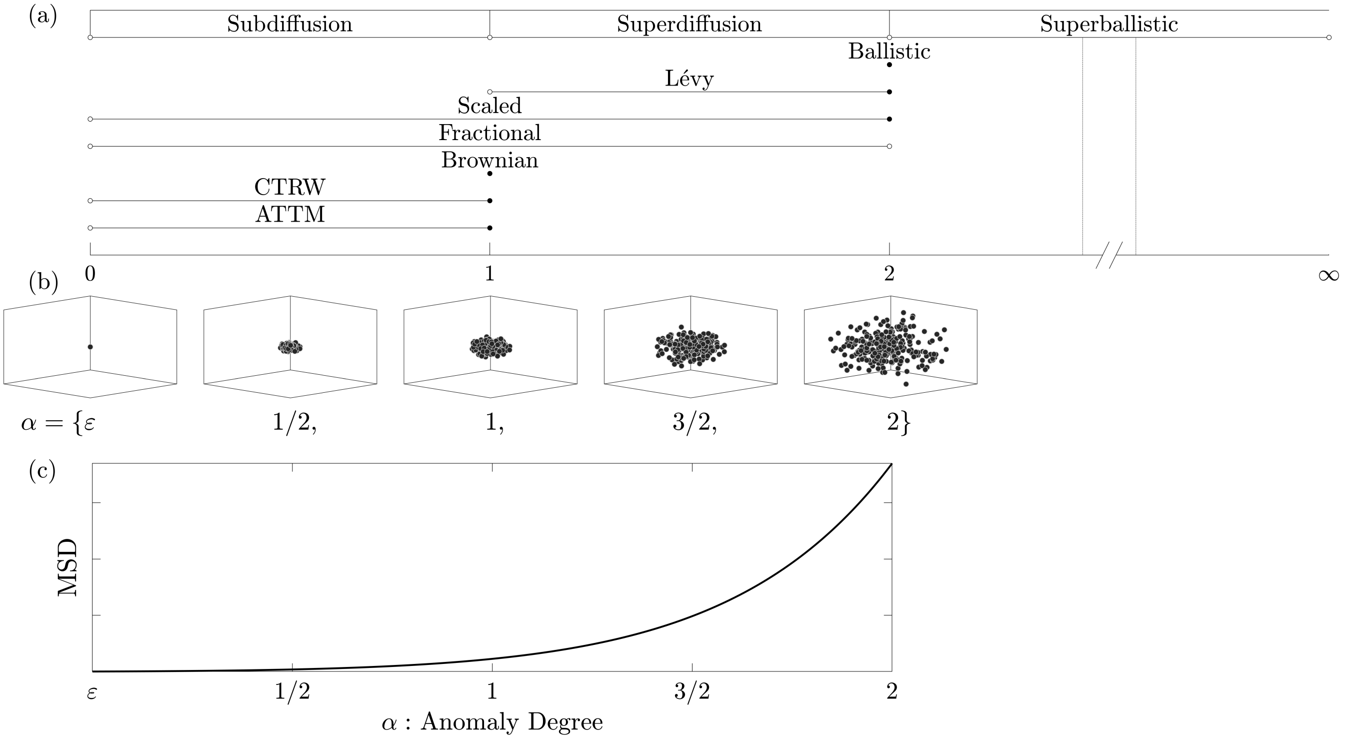

SI Figure 5: Anomalous motion models and the effect of anomalous exponents on scaled Brownian motion (SBM). (a): Here, the anomalous domains and motion models analyzed in this paper are shown within domains of the anomaly degree parameter space. Along the α-number line, hollow and solid dots indicate discontinuous and continuous points, respectively. (b): Motional persistence is visualized by the final positions of 250 diffusing molecules after 10 steps from the origin, given α. Here, ε denotes a very small number to approximate virtually immobile cases. (c) The ensemble-average mean-square displacement's dependence on the anomaly degree α for the ideal data in panel (b).

Above, SI Figure 5 shows diffusive regimes of the anomalous exponent α. In the subdiffusive (*i.e.*, anti-persistent) domain characterized by stochastic motion in crowded, heterogeneous environments, the annealed transient time motion (ATTM) model [9] was developed to reproduce trajectories exhibiting patches of localized BM in spatially disordered media, whereas the continuous-time random walk (CTRW) [10] was designed to simulate diffusion along structural lattices [11] and account for the photoconductivity of amorphous solids [12]. Non-persistent, pure diffusion — Brownian motion — was formulated as a zero-mean Gaussian process with independent, stationary increments [13]. In the persistent superdiffusive domain distinguished by quicker, more directed transits, Lévy distributions [14] were used in devising the spatial Lévy flight [15–17], whose divergent MSD and immediate jumps were remedied by the spatiotemporal Lévy walk (LW) [18–20]; while the former typifies photon transport through disordered optical media [21], the latter portrays patterns from animal foraging [22] to bacterial chemotaxis [23]. Further still, superballistic diffusion is even faster than projectile motion and has been observed in the hydrodynamic flow of electrons through Graphene constrictions.

SI Figure 5 also shows that many anomalous motion models are defined at the Brownian limit ($\alpha = 1$), whereat each model is said to converge to BM [24]. Included in these models are more generalized Gaussian models encompassing a larger range of the anomalous exponent's parameter space: fractional Brownian motion (FBM) yields subdiffusive, Brownian, and superdiffusive motions [25] best describing stochastic propagation through viscoelastic media [2]. Similarly, scaled Brownian motion (SBM) additionally encompasses ballistic diffusion and best represents diffusion coefficients evolving deterministically over time [26, 27] as expected of time-dependent temperature [28] or photobleaching recovery [2].

Tracking Non-Brownian Motion

Here, we show accurate tracking of diffusive trajectories across both motion models and the anomalous exponent α. Below, 749/750 (99.9%) of in-frame ground truth positions are circumscribed within our shaded 98% CI, missing only a single position for subdiffusive CTRW.

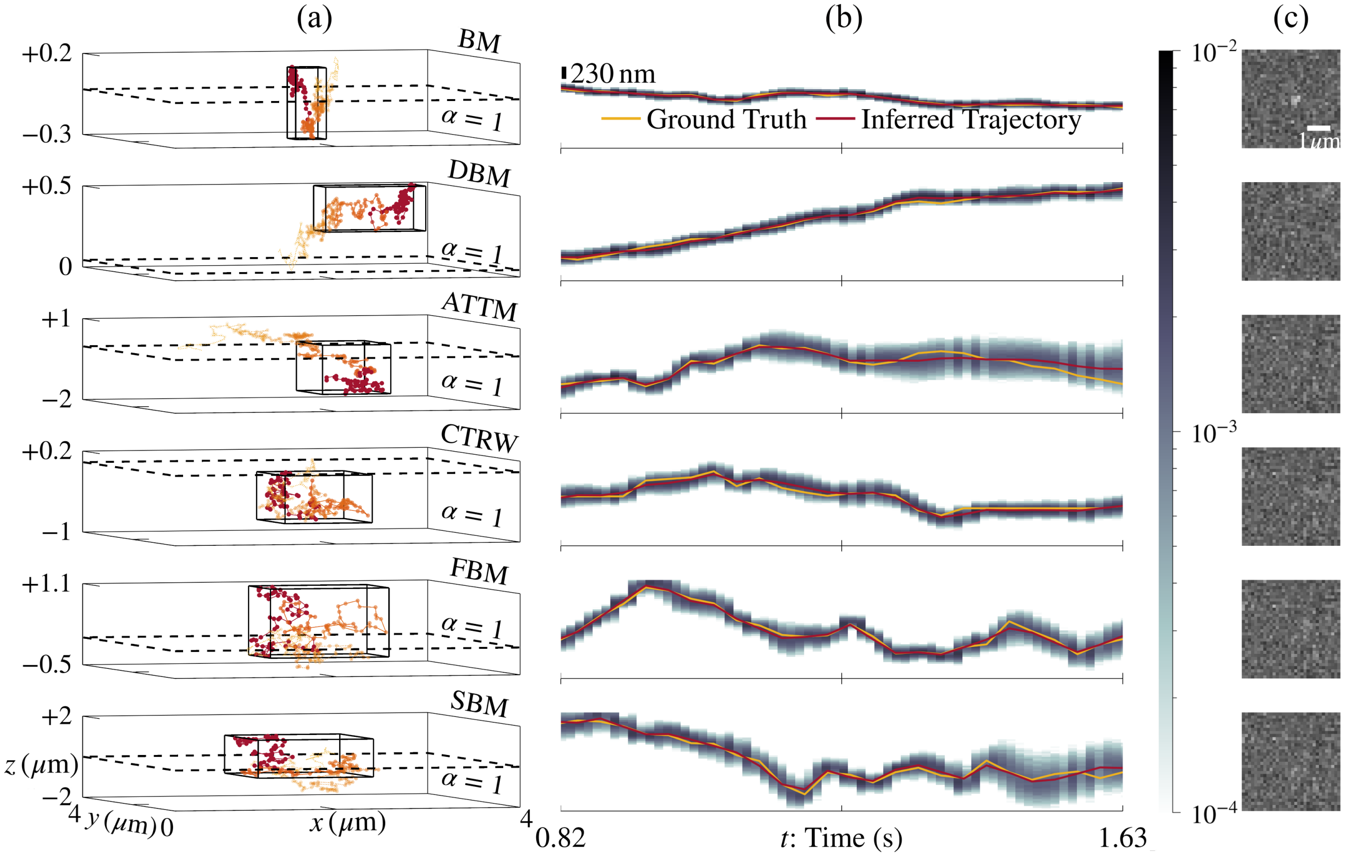

SI Figure 6: A likelihood informed by the BM model accurately tracks particle positions with trajectories generated according to alternate motion models at the Brownian limit. (a) Inferred trajectories $\boldsymbol{R}_{1:N}^{1:K}$ are shown for each motion model with opacity, marker size, and color wavelength increasing with time. A dashed line marks the image plane, whereas the solid three-dimensional box encloses the samples shown in the central panel. (b) Three-dimensional trajectories along the $\hat{\boldsymbol{x}}$ direction are shown for ground truth (gold) and the mean MCMC sample (red); these trajectories are accompanied by a 230 nm scalebar and an associated shading that represents the 98% CI obtained without burn-in over MCMC iterations $i \in [2, 6] \cdot 10^3$. (c) The final image $\boldsymbol{w}_N^{1:P}$ of each motion model is shown with a 1 µm scalebar; these frames have been transposed to align visually with central $x(t)$ plots. The generation of data for this figure is detailed in the **Forward Models for Data Acquisition** within the **Methods** section in the main text. A complete list of assigned measurement parameters is provided in **Table 3** in the main text.

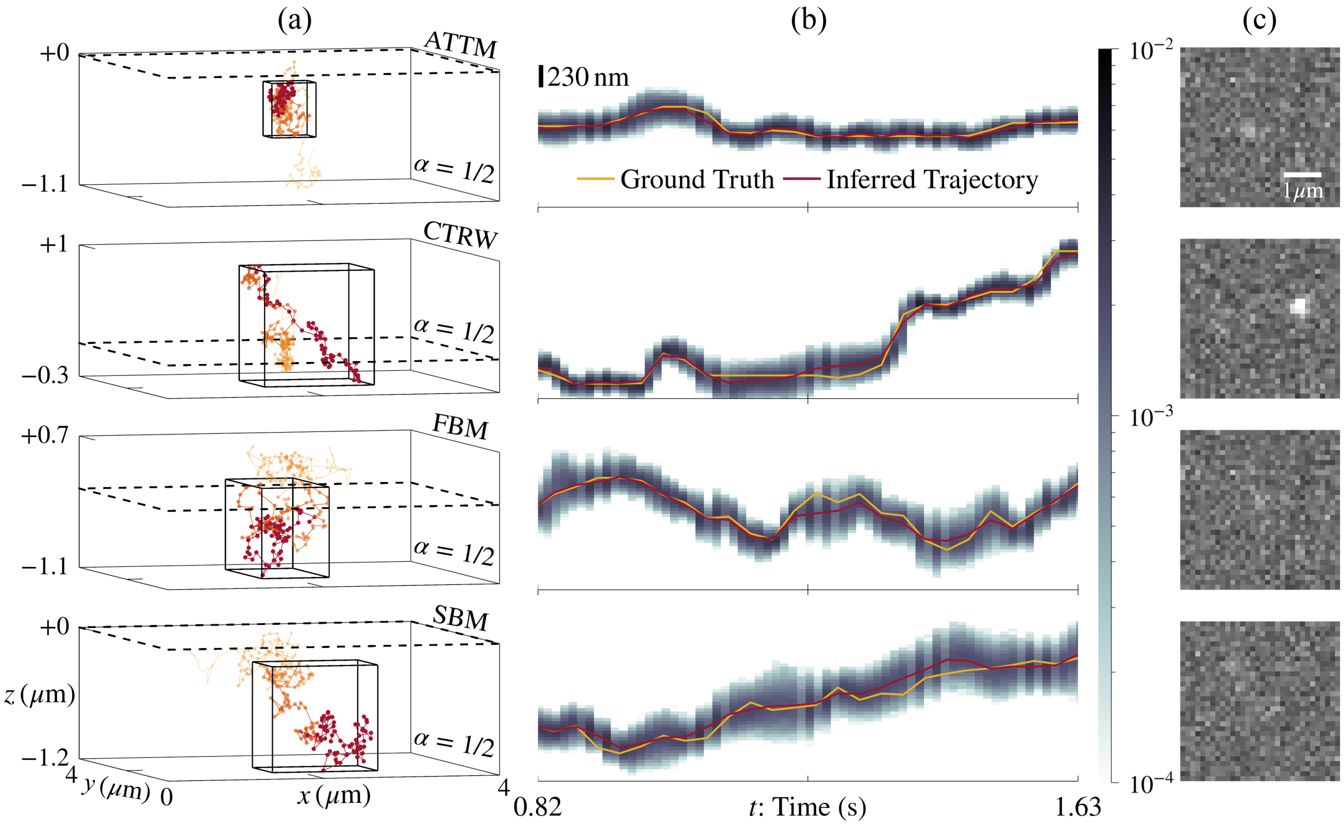

SI Figure 7: A likelihood informed by the BM model accurately tracks particle positions with trajectories generated according to subdiffusive motion models. The figure's layout is identical to SI Figure 6. A complete list of assigned measurement parameters is provided in **Table 3** in the main text.

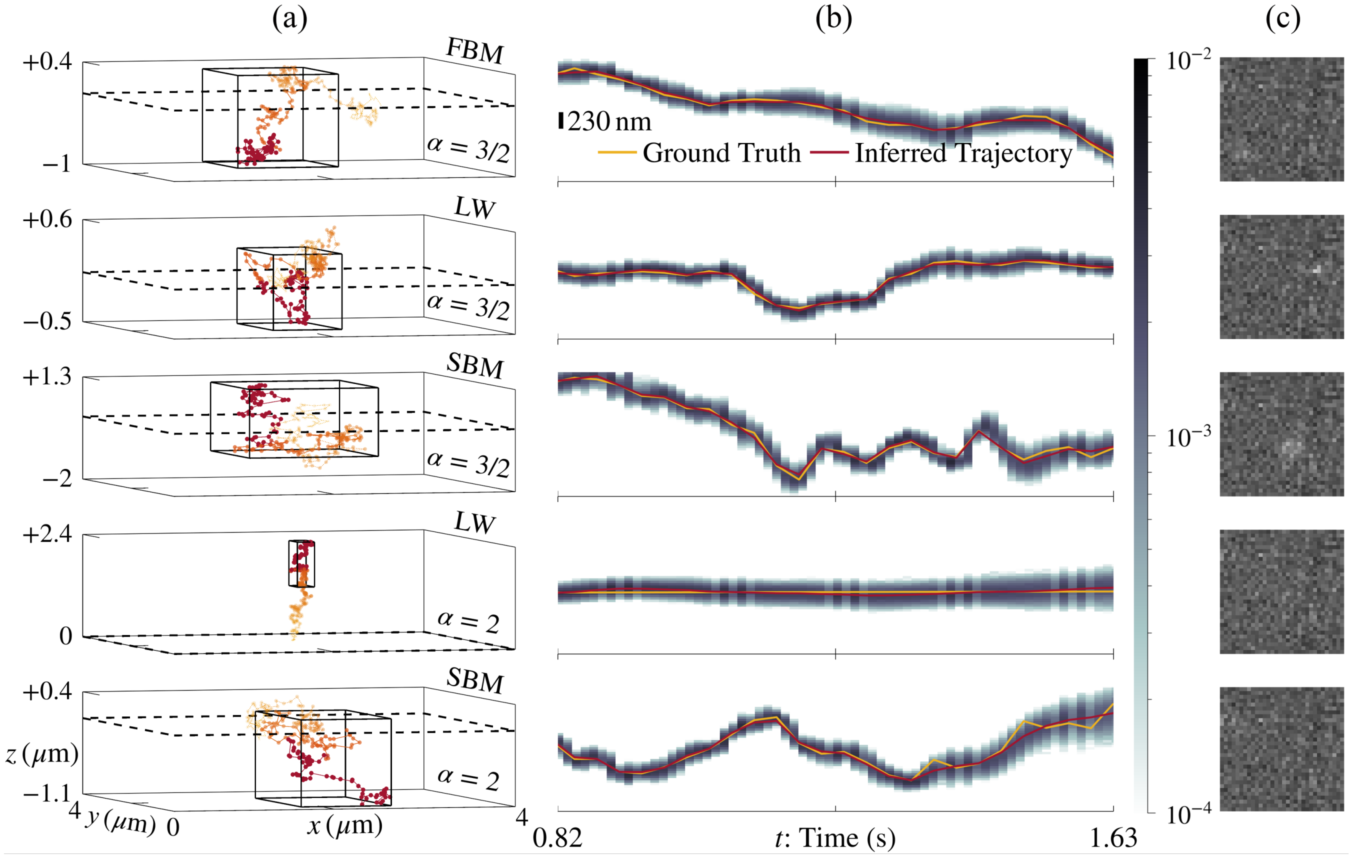

SI Figure 8: A likelihood informed by the BM model accurately tracks particle positions with trajectories generated according to superdiffusive and ballistic motion models. The figure's layout is identical to SI Figure 6. A complete list of assigned measurement parameters is provided in **Table 3** in the main text.

Part V: Supplementary Tables

Method (or Team) Name	Language	Mode of Failure
Teams {A[29,30], C:G[31–35], J:K[36–38], N:O[37,39–43]}	Python	Library Deprecations
AnDi-ELM[44]	MATLAB	Instructionless
NOBIAS[45]	MATLAB	Defunct Example
Teams {G[46], H[47] }		Only Infers α
Teams {A[29,30], D[32,33], H[47], I, K[38] }		1-Dimensional
Gratin[31], *AnomDiffDB*[36,37]		2-Dimensional

Table 3: The majority of methods for decoding anomalous diffusion are either currently unusable or only useful for analyzing one- or two-dimensional trajectories.

References (Supplementary)

1. Krog, J., Jacobsen, L. H., Lund, F. W., Wüstner, D. & Lomholt, M. A. Bayesian Model Selection with Fractional Brownian Motion. *Journal of Statistical Mechanics: Theory and Experiment* **2018,** 093501. `https://dx.doi.org/10.1088/1742-5468/aadb0e` (2018).
2. Thapa, S. *et al.* Bayesian Inference of Scaled Versus Fractional Brownian Motion. *Journal of Physics A: Mathematical and Theoretical* **55,** 194003. `https://dx.doi.org/10.1088/1751-8121/ac60e7` (2022).
3. Brockwell, P. J. & Davis, R. A. *Time Series: Theory and Methods* (Springer New York, NY, 1991).

4. Pressé, S. & Sgouralis, I. *Data Modeling for the Sciences: Applications, Basics, Computations* (Cambridge University Press, 2023).
5. Thompson, R. E., Larson, D. R. & Webb, W. W. Precise Nanometer Localization Analysis for Individual Fluorescent Probes. *Biophysical Journal* **82,** 2775. https://www.sciencedirect.com/science/article/pii/S000634950275618X (2002).
6. Hirsch, M., Wareham, R. J., Martin-Fernandez, M. L., Hobson, M. P. & Rolfe, D. J. A Stochastic Model for Electron Multiplication Charge-Coupled Devices – From Theory to Practice. *PLOS ONE* **8,** 1. https://doi.org/10.1371/journal.pone.0053671 (2013).
7. Huang, F. *et al.* Video-Rate Nanoscopy Using sCMOS Camera–Specific Single-Molecule Localization Algorithms. *Nature Methods* **10,** 653 (2013).
8. Mandracchia, B. *et al.* Fast and Accurate sCMOS Noise Correction for Fluorescence Microscopy. *Nature Communications* **11,** 94. https://doi.org/10.1038/s41467-019-13841-8 (2020).
9. Massignan, P. *et al.* Nonergodic Subdiffusion from Brownian Motion in an Inhomogeneous Medium. *Physical Review Letters* **112,** 150603. https://link.aps.org/doi/10.1103/PhysRevLett.112.150603 (2014).
10. Jeon, J. *et al.* In vivo Anomalous Diffusion and Weak Ergodicity Breaking of Lipid Granules. *Physical Review Letters* **106,** 048103. https://link.aps.org/doi/10.1103/PhysRevLett.106.048103 (2011).
11. Montroll, E. W. & Weiss, G. H. Random Walks on Lattices. II. *Journal of Mathematical Physics* **6,** 167. https://aip.scitation.org/doi/10.1063/1.1704269 (1965).
12. Scher, H. & Montroll, E. W. Anomalous Transit-Time Dispersion in Amorphous Solids. *Physical Review B* **12.** https://link.aps.org/doi/10.1103/PhysRevB.12.2455 (1975).
13. Wiener, N. Differential-Space. *Journal of Mathematics and Physics* **2,** 131. https://onlinelibrary.wiley.com/doi/abs/10.1002/sapm192321131 (1923).
14. Levy, P. *Theorie de l'Addition des Variables Aleatoires* (Gauthier-Villars, 1937).
15. Mandelbrot, B. B. *The Fractal Geometry of Nature* (W. H. Freeman and Company, New York, 1982).
16. Koren, T., Lomholt, M. A., Chechkin, A. V., Klafter, J. & Metzler, R. Leapover Lengths and First Passage Time Statistics for Lévy Flights. *Physical Review Letters* **99,** 160602. https://link.aps.org/doi/10.1103/PhysRevLett.99.160602 (16 2007).
17. Palyulin, V. V., Chechkin, A. V. & Metzler, R. Lévy Flights Do Not Always Optimize Random Blind Search for Sparse Targets. *Proceedings of the National Academy of Sciences* **111,** 2931. https://www.pnas.org/doi/abs/10.1073/pnas.1320424111 (2014).
18. Shlesinger, M. F., Klafter, J. & Wong, Y. M. Random Walks with Infinite Spatial and Temporal Moments. *Journal of Statistical Physics* **27,** 499. https://doi.org/10.1007/BF01011089 (1982).
19. Shlesinger, M. F. & Klafter, J. in *On Growth and Form: Fractal and Non-Fractal Patterns in Physics* (eds Stanley, H. E. & Ostrowsky, N.) 279 (Springer Netherlands, Dordrecht, 1986). https://doi.org/10.1007/978-94-009-5165-5_29.
20. Zaburdaev, V., Denisov, S. & Klafter, J. Lévy Walks. *Reviews of Modern Physics* **87,** 483. https://link.aps.org/doi/10.1103/RevModPhys.87.483 (2 2015).
21. Barthelemy, P., Bertolotti, J. & Wiersma, D. S. A Lévy Flight for Light. *Nature* **453,** 495. https://doi.org/10.1038/nature06948 (2008).
22. Campeau, W., Simons, A. M. & Stevens, B. The Evolutionary Maintenance of Lévy Flight Foraging. *PLOS Computational Biology* **18.** https://doi.org/10.1371/Journal.pcbi.1009490 (2022).
23. Huo, H., He, R., Zhang, R. & Yuan, J. Swimming *Escherichia coli* Cells Explore the Environment by Lévy Walk. *Applied and Environmental Microbiology* **87,** e02429. https://doi.org/10.1128/AEM.02429-20 (2021).
24. Muñoz-Gil, G. *et al.* Objective Comparison of Methods to Decode Anomalous Diffusion. *Nature Communications* **12.** https://doi.org/10.1038/s41467-021-26320-w (2021).
25. Mandelbrot, B. B. & Van Ness, J. W. Fractional Brownian Motions, Fractional Noises and Applications. *SIAM Review* **10** (1968).
26. Lim, S. C. & Muniandy, S. V. Self-similar Gaussian processes for modeling anomalous diffusion. *Physical Review E* **66,** 021114. https://link.aps.org/doi/10.1103/PhysRevE.66.021114 (2 2002).
27. Jeon, J.-H., Chechkin, A. V. & Metzler, R. Scaled Brownian Motion: A Paradoxical Process with a Time Dependent Diffusivity for the Description of Anomalous Diffusion. *Physical Chemistry Chemical Physics* **16.** http://dx.doi.org/10.1039/C4CP02019G (2014).
28. Bodrova, A. S. *et al.* Underdamped Scaled Brownian Motion: (Non-)Existence of the Overdamped Limit in Anomalous Diffusion. *Scientific Reports* **6,** 30520. https://doi.org/10.1038/srep30520 (2016).
29. Wolpert, D. H. Stacked Generalization. *Neural Networks* **5,** 241. https://www.sciencedirect.com/science/article/pii/S0893608005800231 (1992).

30. Muñoz-Gil, G. *et al.* Phase Separation of Tunable Biomolecular Condensates Predicted by an Interacting Particle Model. *bioRxiv.* https://www.biorxiv.org/content/early/2022/01/25/2020.09.09.289876 (2022).
31. Verdier, H. *et al.* Learning Physical Properties of Anomalous Random Walks Using Graph Neural Networks. *Journal of Physics A: Mathematical and Theoretical* **54,** 234001. http://dx.doi.org/10.1088/1751-8121/abfa45 (2021).
32. He, K., Zhang, X., Ren, S. & Sun, J. *Deep Residual Learning for Image Recognition* in *2016 IEEE Conference on Computer Vision and Pattern Recognition (CVPR)* (2016), 770.
33. Chen, T. & Guestrin, C. *XGBoost: A Scalable Tree Boosting System* in *Proceedings of the 22nd ACM SIGKDD International Conference on Knowledge Discovery and Data Mining* (Association for Computing Machinery, San Francisco, California, USA, 2016), 785. ISBN: 9781450342322. https://doi.org/10.1145/2939672.2939785.
34. Arts, M., Smal, I., Paul, M. W., Wyman, C. & Meijering, E. Particle Mobility Analysis Using Deep Learning and the Moment Scaling Spectrum. *Scientific Reports* **9,** 17160. https://doi.org/10.1038/s41598-019-53663-8 (2019).
35. Argun, A., Volpe, G. & Bo, S. Classification, Inference and Segmentation of Anomalous Diffusion with Recurrent Neural Networks. *Journal of Physics A: Mathematical and Theoretical* **54,** 294003. https://dx.doi.org/10.1088/1751-8121/ac070a (2021).
36. Bai, S., Kolter, J. Z. & Koltun, V. An Empirical Evaluation of Generic Convolutional and Recurrent Networks for Sequence Modeling. *Computing Research Repository in ArXiv.* http://arxiv.org/abs/1803.01271 (2018).
37. Granik, N. *et al.* Single-Particle Diffusion Characterization by Deep Learning. *Biophysical Journal* **117,** 185. https://www.sciencedirect.com/science/article/pii/S0006349519305041 (2019).
38. Aghion, E., Meyer, P. G., Adlakha, V., Kantz, H. & Bassler, K. E. Moses, Noah and Joseph effects in Lévy Walks. *New Journal of Physics* **23,** 023002. https://dx.doi.org/10.1088/1367-2630/abd43c (2021).
39. Lines, J., Taylor, S. & Bagnall, A. Time Series Classification with HIVE-COTE: The Hierarchical Vote Collective of Transformation-Based Ensembles. *ACM Transactions on Knowledge Discovery from Data* **12.** https://doi.org/10.1145/3182382 (2018).
40. Le Nguyen, T., Gsponer, S., Ilie, I., O'Reilly, M. & Ifrim, G. Interpretable Time Series Classification Using Linear Models and Multi-Resolution Multi-Domain Symbolic Representations. *Data Mining and Knowledge Discovery* **33,** 1183. ISSN: 1573-756X. https://doi.org/10.1007/s10618-019-00633-3 (2019).
41. Kowalek, P., Loch-Olszewska, H. & Szwabi ński, J. Classification of Diffusion Modes in Single-Particle Tracking Data: Feature-Based Versus Deep-Learning Approach. *Physical Review E* **100,** 032410. https://link.aps.org/doi/10.1103/PhysRevE.100.032410 (3 2019).
42. Loch-Olszewska, H. & Szwabiński, J. Impact of Feature Choice on Machine Learning Classification of Fractional Anomalous Diffusion. *Entropy* **22.** https://www.mdpi.com/1099-4300/22/12/1436 (2020).
43. Janczura, J., Kowalek, P., Loch-Olszewska, H., Szwabi ński, J. & Weron, A. Classification of Particle Trajectories in Living Cells: Machine Learning Versus Statistical Testing Hypothesis for Fractional Anomalous Diffusion. *Physical Review E* **102,** 032402. https://link.aps.org/doi/10.1103/PhysRevE.102.032402 (3 2020).
44. Manzo, C. Extreme Learning Machine for the Characterization of Anomalous Diffusion from Single Trajectories (AnDi-ELM). *Journal of Physics A: Mathematical and Theoretical* **54,** 334002. https://dx.doi.org/10.1088/1751-8121/ac13dd (2021).
45. Chen, Z., Geffroy, L. & Biteen, J. S. NOBIAS: Analyzing Anomalous Diffusion in Single-Molecule Tracks With Nonparametric Bayesian Inference. *Frontiers in Bioinformatics* **1.** https://www.frontiersin.org/journals/bioinformatics/articles/10.3389/fbinf.2021.742073 (2021).
46. Li, D., Yao, Q. & Huang, Z. WaveNet-Based Deep Neural Networks for the Characterization of Anomalous Diffusion (WADNet). *Journal of Physics A: Mathematical and Theoretical* **54,** 404003. https://dx.doi.org/10.1088/1751-8121/ac219c (2021).
47. Donahue, J. *et al.* Long-Term Recurrent Convolutional Networks for Visual Recognition and Description. *IEEE Transactions on Pattern Analysis and Machine Intelligence* **39,** 677 (2017).
